# Fluorescence imaging assisted precise assessment of the depth of myometrial invasion in endometrial cancer lesions

**DOI:** 10.1002/ctm2.70309

**Published:** 2025-05-07

**Authors:** Qiaojun Qu, Huilong Nie, Shuang Hou, Xiaoyong Guo, Feng Wang, Hua Yang, Shangqiu Chen, Panxia Deng, Zhenhua Hu, Jie Tian

**Affiliations:** ^1^ Department of Radiology First Hospital of Shanxi Medical University Taiyuan China; ^2^ CAS Key Laboratory of Molecular Imaging Beijing Key Laboratory of Molecular Imaging Institute of Automation Chinese Academy of Sciences Beijing China; ^3^ Department of Gynecology The Fifth Affiliated Hospital of Sun Yat‐sen University Zhuhai China; ^4^ Key Laboratory of Carcinogenesis and Translational Research Department of Gastrointestinal Cancer Center Ward I, Peking University Cancer Hospital & Institute Beijing China; ^5^ School of Artificial Intelligence University of Chinese Academy of Sciences Beijing China; ^6^ National Key Laboratory of Kidney Diseases Beijing China; ^7^ Key Laboratory of Big Data‐Based Precision Medicine of Ministry of Industry and Information Technology School of Engineering Medicine Beihang University Beijing China; ^8^ Engineering Research Center of Molecular and Neuro Imaging of Ministry of Education School of Life Science and Technology Xidian University Xi'an China

1

Dear Editor,

Accurate evaluation of myometrial invasion depth (MID) during endometrial cancer surgery is crucial for determining the extent of lymph node dissection and prognosis.^1^ However, both preoperative magnetic resonance imaging (MRI) and intraoperative visual observation have limitations in MID evaluation.^2^, ^3^, ^4^ Hence, there is an urgent demand for novel imaging techniques to aid surgeons in accurately determining MID during surgical procedures. The fluorescence imaging within the near‐infrared II (NIR‐II, 1000–1700 nm) spectral window shows significant promise for medical applications.^5^, ^6^, ^7^, ^8^ This study aimed to investigate whether this approach could serve as a reliable method for the precise assessment of MID.

Eight patients with endometrial cancer were enrolled in this study between November 2021 and May 2022. Inclusion criteria comprised individuals between the ages of 18 and 75 years, who had recently received a histologically confirmed diagnosis of endometrial cancer, preoperative International Federation of Gynecology and Obstetrics (FIGO) stage I (endometrial cancer confined to the uterine corpus) and normal liver and kidney functions. Exclusion criteria encompassed a history of uterine surgery and preoperative chemoradiotherapy. Written informed consent was obtained from all participants.

The study protocol was shown in Figure S1. All enrolled patients underwent preoperative MRI and hysteroscopic biopsy to confirm the diagnosis of endometrial cancer. Patients were administered intravenous infusion of the fluorescent dye indocyanine green (ICG) at a dosage of 5 mg/kg 24 h before surgery, followed by total hysterectomy with bilateral salpingo‐oophorectomy (TH/BSO). To delineate the extent of lymph node dissection, a ‘Y’‐shaped incision was made in the excised uterus for visual assessment of the size, scope and MID of the endometrial cancer. Subsequently, NIR‐II fluorescence imaging and visible‐light imaging (VLI) of the excised uterus were conducted. The fluorescence tumour‐to‐background ratio (TBR) was quantified, and various regions of interest, including the cancer lesions, normal myometrium and lesions adjacent to normal myometrium, were sampled for microscopic imaging. Finally, MID was assessed using NIR‐II fluorescence imaging. The ICG dosage and timing were based on previous study demonstrating optimal TBR at this dose and timepoint.^9^

A specialised NIR‐II fluorescence imaging system was meticulously assembled for precise NIR‐II fluorescence imaging. This comprehensive setup consisted of four essential components: the NIR‐II imaging instrument, laser excitation device, water cooling system and a computer monitor. At the core of the NIR‐II imaging instrument were an InGaAs camera (Cheetah‐640‐CL; Vision Smart) and a high‐sensitivity lens (EF 24–70 mm F/2.8L II USM; Canon). To optimise the collection of NIR‐II fluorescence signals, an optical filter (1000 nm LP, FEL1000; Thorlabs) was seamlessly connected to the lens through an adapter. The excitation device included a 792 nm wavelength laser, an optical fibre and a beam expander. The imaging setup maintained a working distance of approximately 50 cm.

Quantitative data in this study were expressed as mean ± standard deviation. The TBR was calculated as the mean fluorescence signal intensity of the lesion by that of the surrounding normal myometrium. A TBR value greater than 2 indicates fluorescent.^9^, ^10^ The MID was defined as the ratio of invaded myometrial thickness to total myometrial thickness. A ratio≥1/2 indicates deep myometrial invasion. The association of clinicopathological features with TBR was assessed using Spearman rank‐order correlation. A *p* value less than .05 was considered to indicate statistical significance.

The age range of the eight patients diagnosed with endometrial cancer was between 44 and 75 years. The mean body mass index (BMI) of the patients was 24.7 ± 2.6. All pathological types of endometrial cancer were endometrioid adenocarcinomas. The histological grade was recorded: four cases were classified as grade 1, one as grade 2 and three as grade 3. Notably, all endometrial cancer lesions exhibited fluorescence (Figures 1, 2 and S2C), with an average TBR of 4.0 ± 2.4. Detailed patient characteristics and lesions data are presented in Table S1. Correlation analysis revealed that the TBR was not related to age, BMI or histological grade (all *p *> .05). In addition, all uterine fibroids were non‐fluorescent (Figures 1C,E and S2C). None of the patients had any related adverse reactions within two weeks of ICG injection.

**FIGURE 1:**
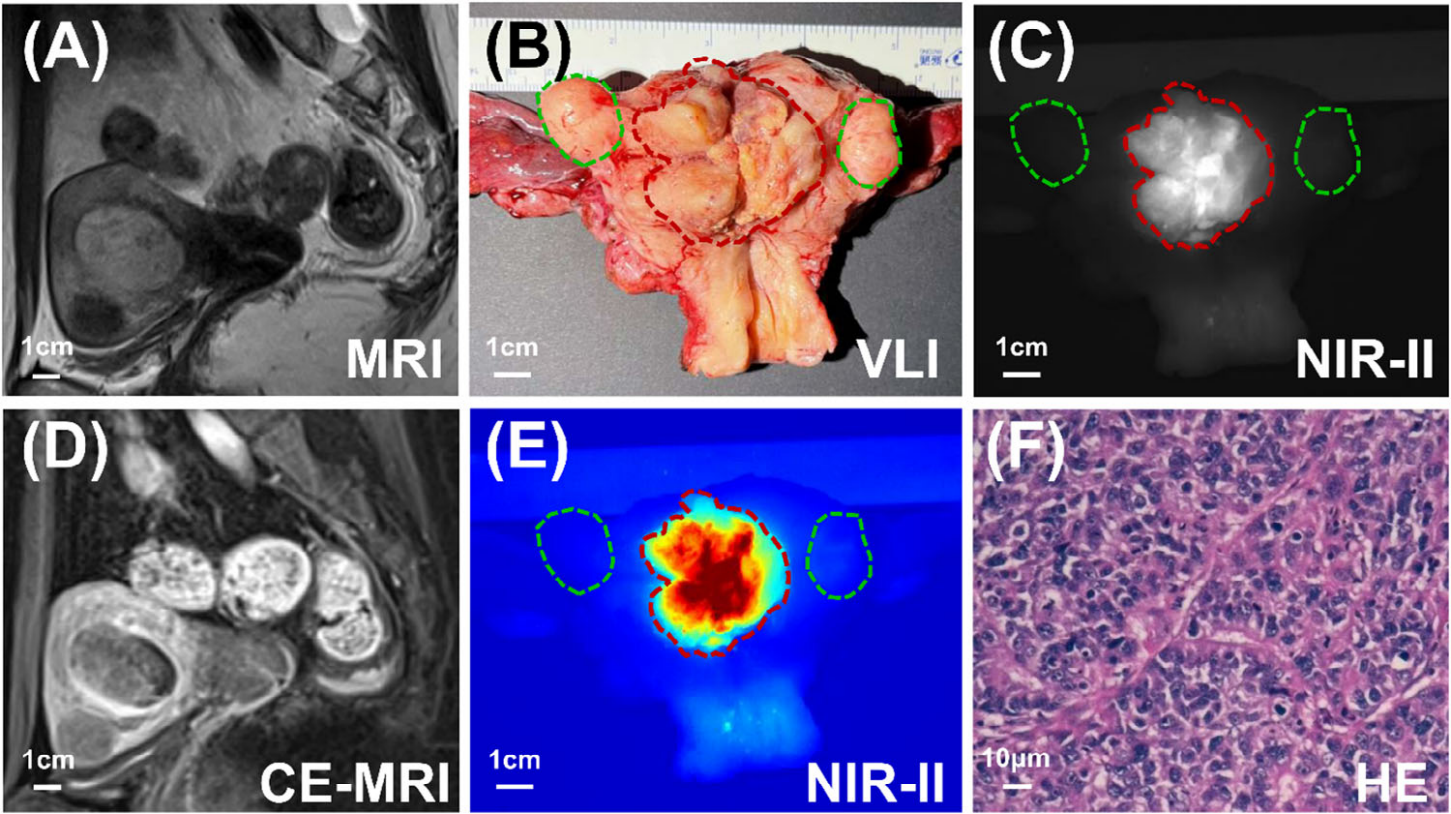
Representative images of NIR‐II fluorescence imaging of the endometrial cancer lesion. (A and D) MRI and contrast‐enhanced magnetic resonance images (CE‐MRI) showed the uterine cavity was filled with endometrial cancer lesion. (B) The endometrial cancer lesion (red dotted line) and uterine fibroids (green dotted line) was visualised by VLI after the intraoperative ‘Y’‐shaped incision of the excised uterus. (C and E) The endometrial cancer lesion (red dotted line) was highlighted by NIR‐II fluorescence imaging, while uterine fibroids (green dotted line) were not highlighted. (F) Endometrial cancer lesion was confirmed by HE staining.

**FIGURE 2:**
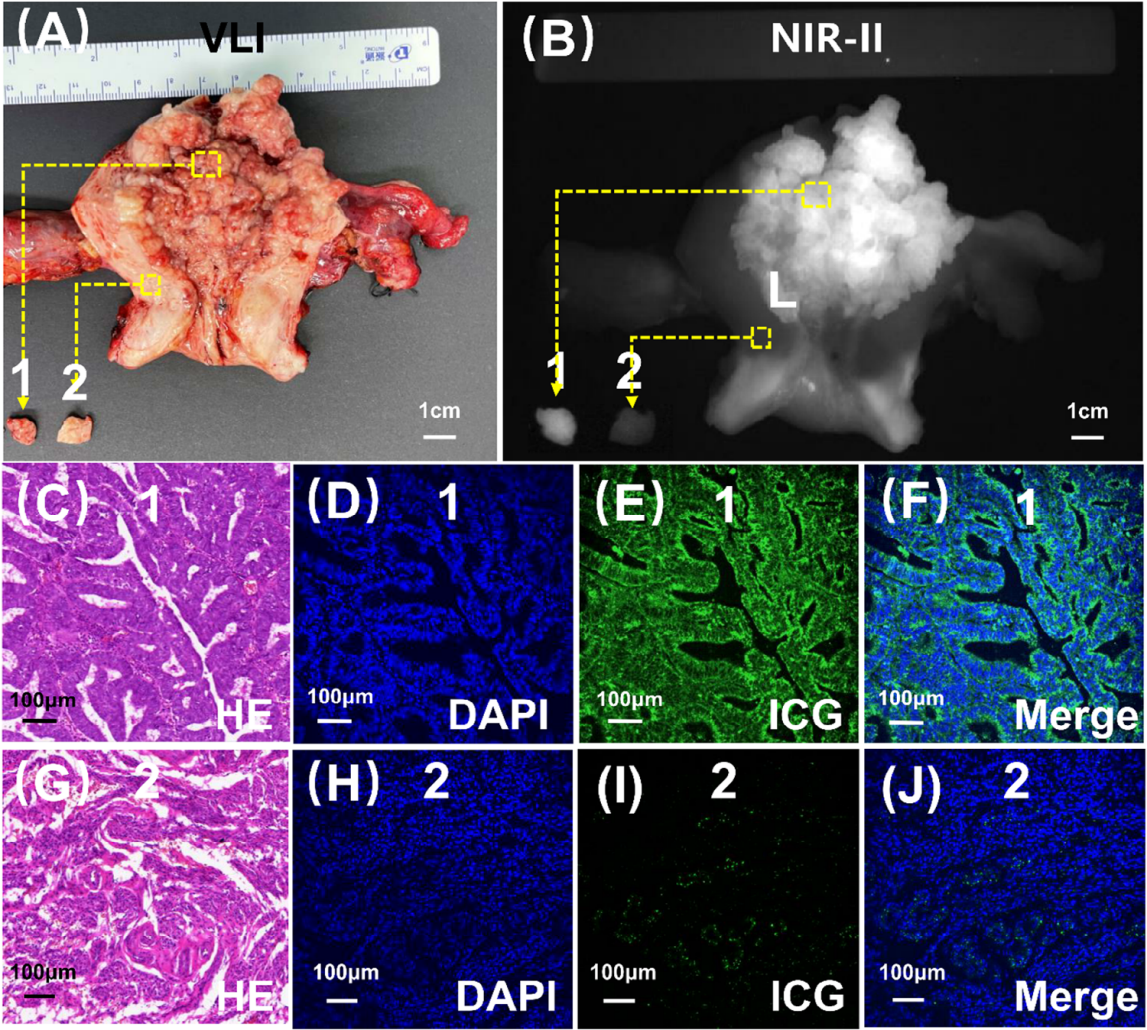
Representative fluorescence microscopic imaging of the endometrial cancer lesion and normal myometrium. (A and B) Endometrial cancer lesion (No. 1) and normal myometrium (No. 2) were sampled respectively from the excised uterus. (C–F) HE staining images, DAPI fluorescence microscopic imaging, ICG fluorescence microscopic imaging and ICG and DAPI overlay fluorescence microscopic imaging of No. 1 tissue. (G–J) HE staining images, DAPI fluorescence microscopic imaging, ICG fluorescence microscopic imaging and ICG and DAPI overlay fluorescence microscopic imaging of No. 2 tissue.

Subsequently, we conducted microscopic verification. Our observations revealed a substantial accumulation of ICG molecules within the tumour (Figure 2B,C–F), contrasting with the limited distribution of ICG in the myometrium (Figure 2B,G–J). This differential distribution pattern accounted for the distinct and clear visualisation of endometrial cancer lesions using NIR‐II fluorescence imaging.

To delve deeper into the correlation between the fluorescence border and the tumour pathological border, samples of endometrial cancer lesions along with the surrounding normal myometrium were obtained and subjected to section analysis. Visually, margins were indiscernible (Figure 3A), yet NIR‐II imaging effectively delineated the boundary (Figure 3B,C). Comparison of pathology and microscopic fluorescence images revealed a strong alignment between the fluorescence edge and the tumour pathological edge, as depicted in Figure 3D–G. These findings offer substantial theoretical backing for the utilisation of NIR‐II fluorescence imaging with ICG in the assessment of MID.

**FIGURE 3:**
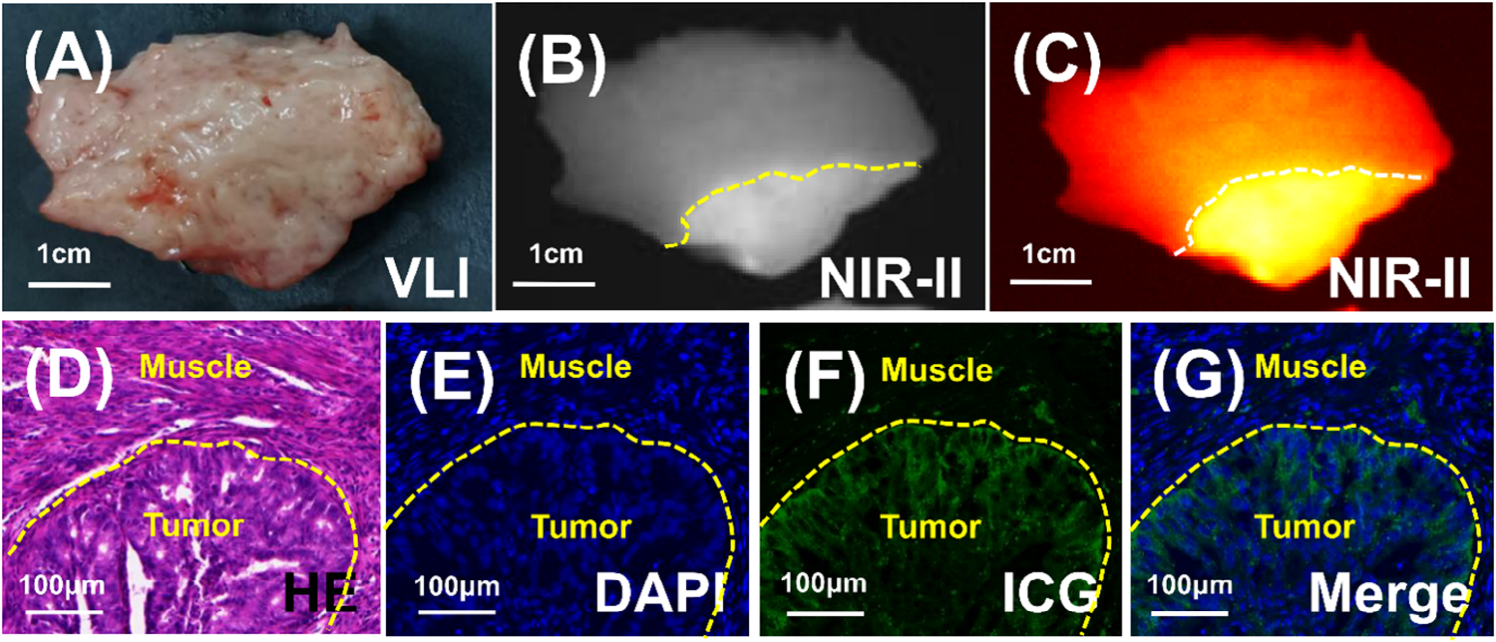
Representative fluorescence images of endometrial cancer lesions accompanied by normal surrounding myometrium. (A–C) The endometrial cancer margins were not detectable in VLI, while it (dotted line) was clearly visualised by NIR‐II fluorescence imaging. (D–G) Further corresponding HE staining images, ICG fluorescence microscopic imaging, DAPI fluorescence microscopic imaging and ICG and DAPI overlay fluorescence microscopic imaging showed that the fluorescence edge and tumour pathological edge have good consistency.

Upon confirming the efficacy of NIR‐II fluorescence imaging in delineating cancer boundaries, we proceeded to employ this technique for MID assessment in endometrial cancer cases. Initially, we imaged the lesion (Figure 4A–C) and identified the region of greatest invasion using NIR‐II fluorescence imaging (Figures 4D–F and S3A–C). Subsequently, we measured the thickness of the uninvaded and completely normal myometrium in the vicinity (Figures 4D–F and S3A–C). This process enabled us to calculate the MID thickness and determine if it exceeded half the myometrial thickness. To evaluate the accuracy of MID assessment, we utilised postoperative pathological analysis of gross specimens as the gold standard. In comparison, the accuracy rates for MID assessment using intraoperative MRI and intraoperative visual observation were 50 and 62.5%, respectively. Remarkably, the accuracy of NIR‐II fluorescence imaging technology reached 100%. Detailed results are presented in Table S2. These findings provide preliminary evidence indicating that NIR‐II fluorescence imaging could serve as a valuable new tool for MID assessment.

**FIGURE 4:**
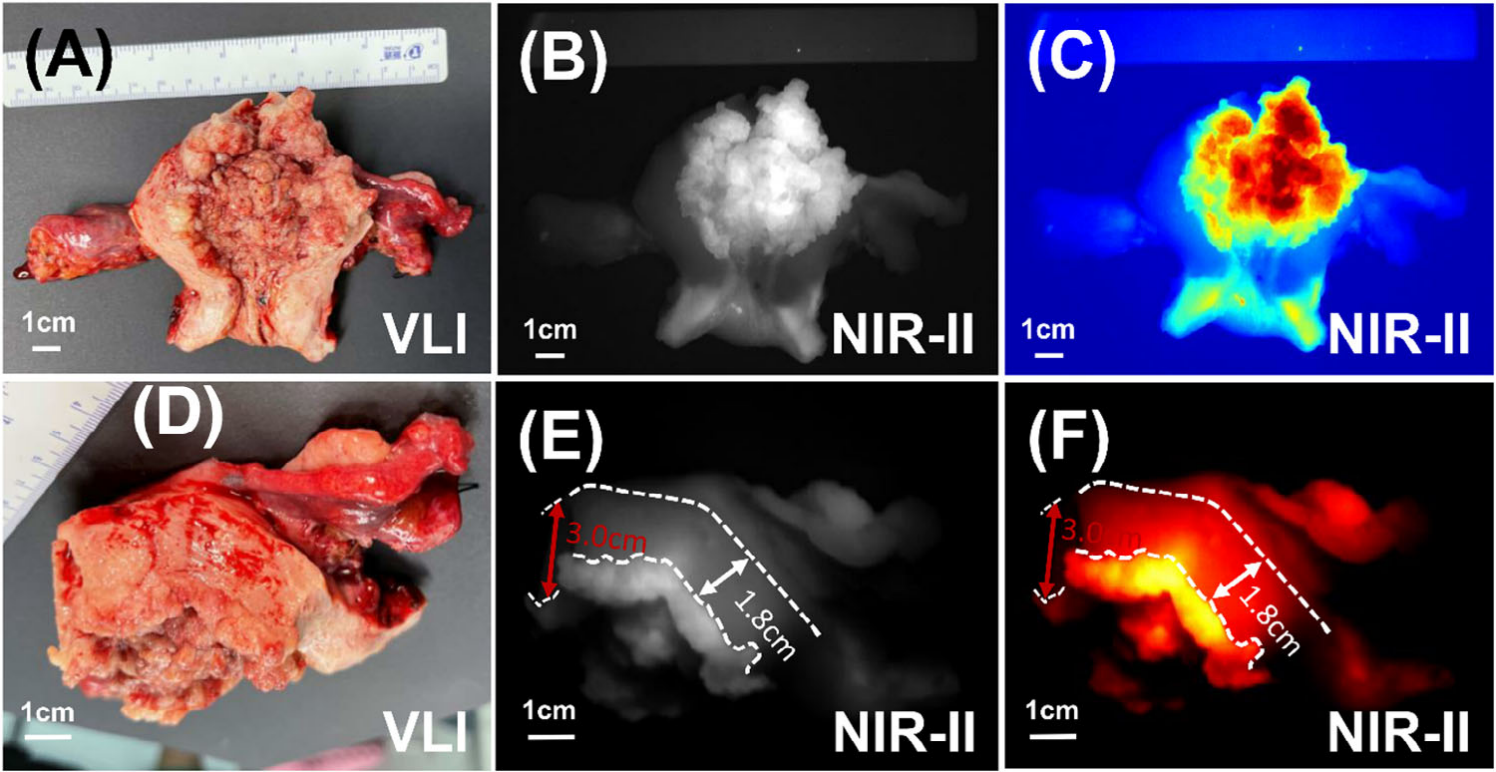
Assessment of MID by NIR‐II fluorescence imaging. (A–C) The endometrial cancer lesions were visualised by VLI and NIR‐II fluorescence imaging. (D–F) Uninvaded and full normal myometrium thickness was measured.

In conclusion, this study demonstrated that NIR‐II fluorescence imaging with ICG effectively delineated endometrial cancer lesions, providing a reliable method for the precise assessment of MID. Notably, none of the uterine fibroids in our study exhibited fluorescence, indicating that this technique could be utilised to determine MID in patients with endometrial cancer and coexisting uterine fibroids. This innovative approach holds promise in assisting surgeons to precisely define the extent of lymph node dissection, thereby contributing to improved patient prognoses.

## AUTHOR CONTRIBUTIONS

All authors contributed to the study conception and design. Material preparation, data collection and analysis were performed by Qiaojun Qu, Huilong Nie, Shuang Hou and Xiaoyong Guo. Technical support was provided by Feng Wang, Hua Yang, Shangqiu Chen and Panxia Deng. The first draft of the manuscript was written by Qiaojun Qu. Revision of the draft was performed by Zhenhua Hu and Jie Tian. All authors read and approved the final manuscript.

## CONFLICT OF INTEREST STATEMENT

The authors declare no conflicts of interest.

## FUNDING INFORMATION

This study was supported by the National Natural Science Foundation of China (NSFC) (62425116, 82427807, 62027901, 92359301, 92459304 and 81227901) and Shanxi Province Science Foundation for Youths (202303021212319).

## ETHICS STATEMENT

This study was approved by the Committee of the Fifth Affiliated Hospital, Sun Yat‐sen University (approval code: 2022‐K72‐1). This study registered at the Chinese Clinical Trial Registration Center (ChiCTR2200061364).

## CONSENT

Informed consent was obtained from the subjects prior to participating in the study.

## Supporting information



Supporting information

## Data Availability

Data can be provided upon reasonable request by contacting the corresponding author.
