# Supplementary material for: Fluorescence imaging assisted precise assessment of the depth of myometrial invasion in endometrial cancer lesions
Source: Clin Transl Med. 2025 May 7;15(5):e70309. doi: 10.1002/ctm2.70309 (PMC12056491; doi:10.1002/ctm2.70309)
Supplement: Supplementary file 1 — Supporting information [file CTM2-15-e70309-s001.docx]

**——Supplementary Materials——**

Fluorescence Imaging Assisted Precise Assessment of the Depth of Myometrial Invasion in Endometrial Cancer Lesions

**Authors**

Qiaojun Qu^1, 2, #^, Huilong Nie^3, #^, Shuang Hou^3, #^, Xiaoyong Guo^4, #^, Feng Wang^3^, Hua Yang^3^, Shangqiu Chen^3^, Panxia Deng^3^, Zhenhua Hu^2, 5, 6, *^, Jie Tian^2, 5, 6, 7, 8, *^

**Affiliations**

^1^Department of Radiology, First Hospital of Shanxi Medical University, Taiyuan, China

^2^CAS Key Laboratory of Molecular Imaging, Beijing Key Laboratory of Molecular Imaging, Institute of Automation, Chinese Academy of Sciences, Beijing, China

^3^Department of Gynecology, The Fifth Affiliated Hospital of Sun Yat-sen University, Zhuhai, China

^4^Key Laboratory of Carcinogenesis and Translational Research, Department of Gastrointestinal Cancer Center, Ward I, Peking University Cancer Hospital & Institute, Beijing, China

^5^School of Artificial Intelligence, University of Chinese Academy of Sciences, Beijing, China

^6^National Key Laboratory of Kidney Diseases, Beijing, China

^7^Key Laboratory of Big Data-Based Precision Medicine of Ministry of Industry and Information Technology, School of Engineering Medicine, Beihang University, Beijing, China

^8^Engineering Research Center of Molecular and Neuro Imaging of Ministry of Education, School of Life Science and Technology, Xidian University, Xi’an, China

**#These authors contributed equally to this work.**

***Correspondence to:**

Zhenhua Hu, Ph.D.

Chinese Academy of Sciences Key Laboratory of Molecular Imaging

95 Zhongguancun East Road, Beijing, China, 100190

E-mail: zhenhua.hu@ia.ac.cn

Jie Tian, Ph.D.

Chinese Academy of Sciences Key Laboratory of Molecular Imaging

95 Zhongguancun East Road, Beijing, China, 100190

E-mail: jie.tian@ia.ac.cn


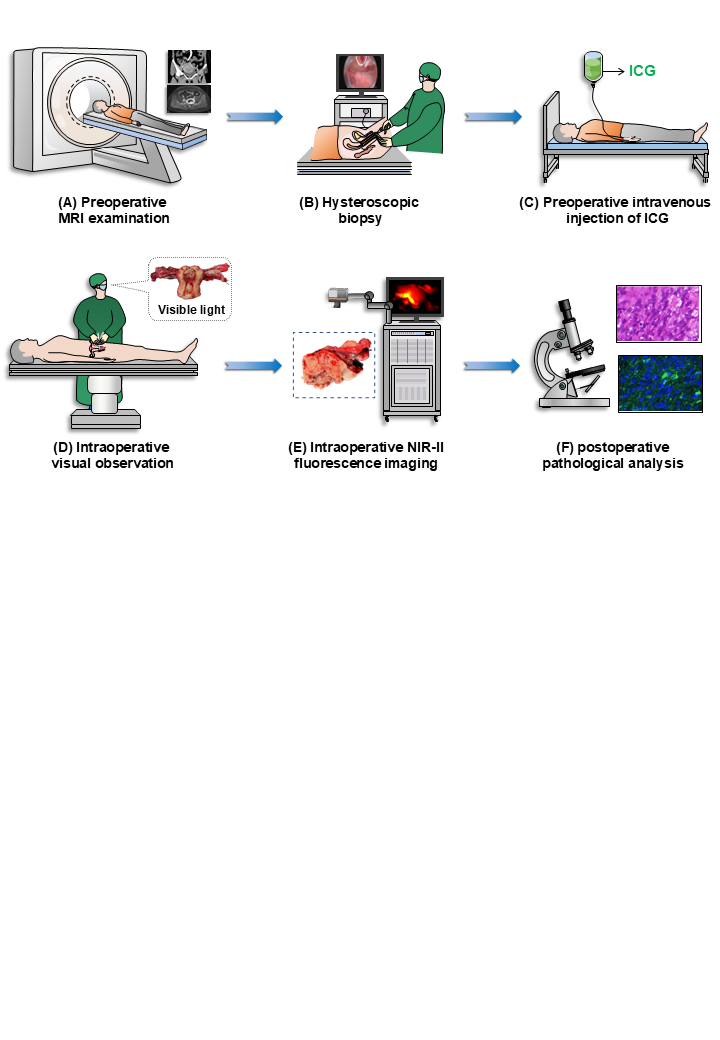


**FIGURE S1**

The protocol of this study. (A, B) Endometrial cancer patients diagnosed by preoperative MRI and hysteroscopic biopsy were enrolled in this clinical trial. (C) ICG with a dose of 5 mg/kg was administrated intravenously 24 h before surgery. (D) In order to determine the scope of lymph node dissection, a "Y"-shaped incision was made in the excised uterus for visual assessment of the size, scope, and MID of the endometrial cancer. (E) NIR-II fluorescence imaging was performed on the excised uterus. (F) Pathological analysis of different tissues was conducted.


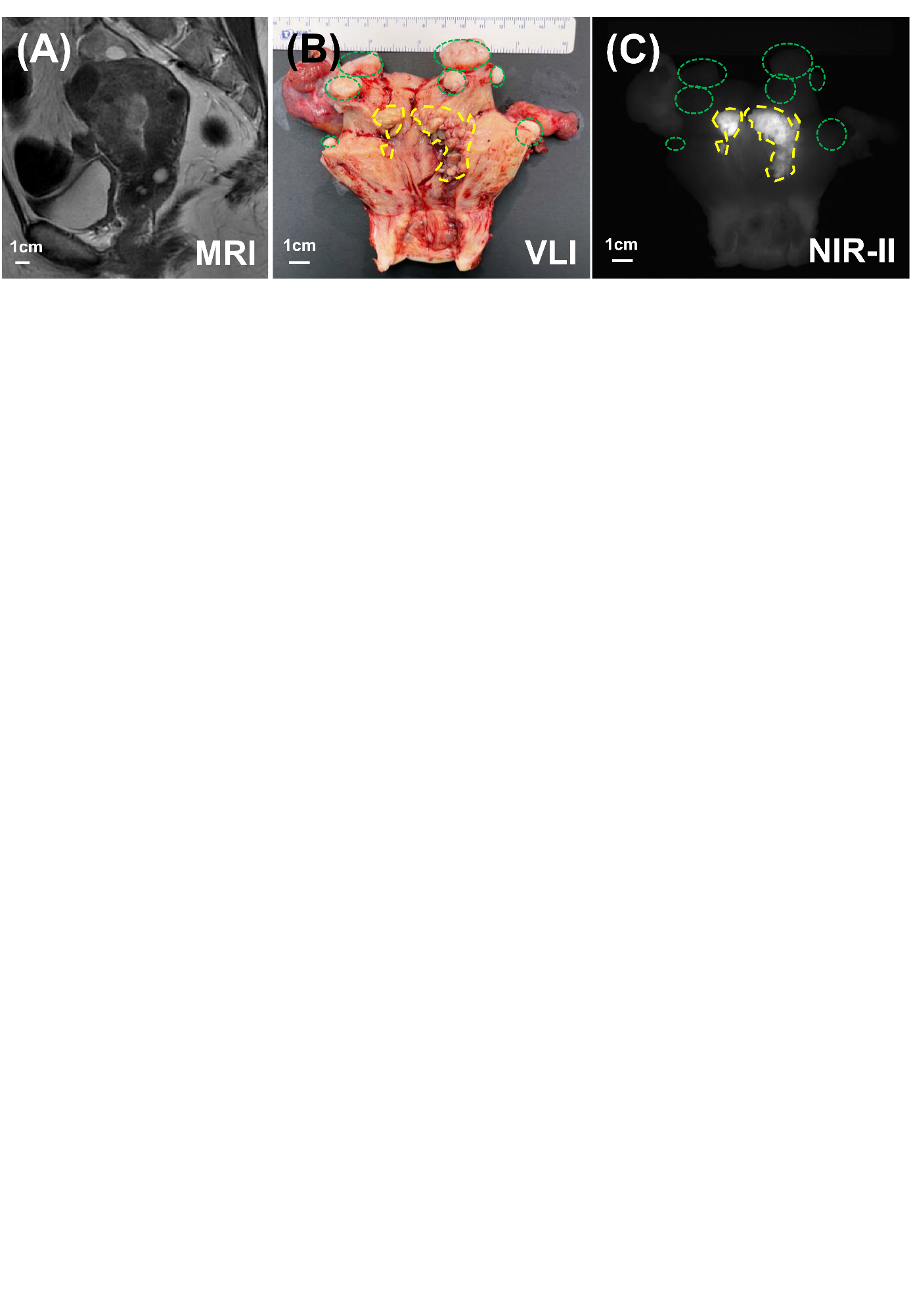


**FIGURE S2**

Fluorescence imaging effect of different tumors. (A-C) MRI, VLI and NIR-II fluorescence imaging of uterine fibroids (green dotted line) and endometrial cancer (yellow dotted line).


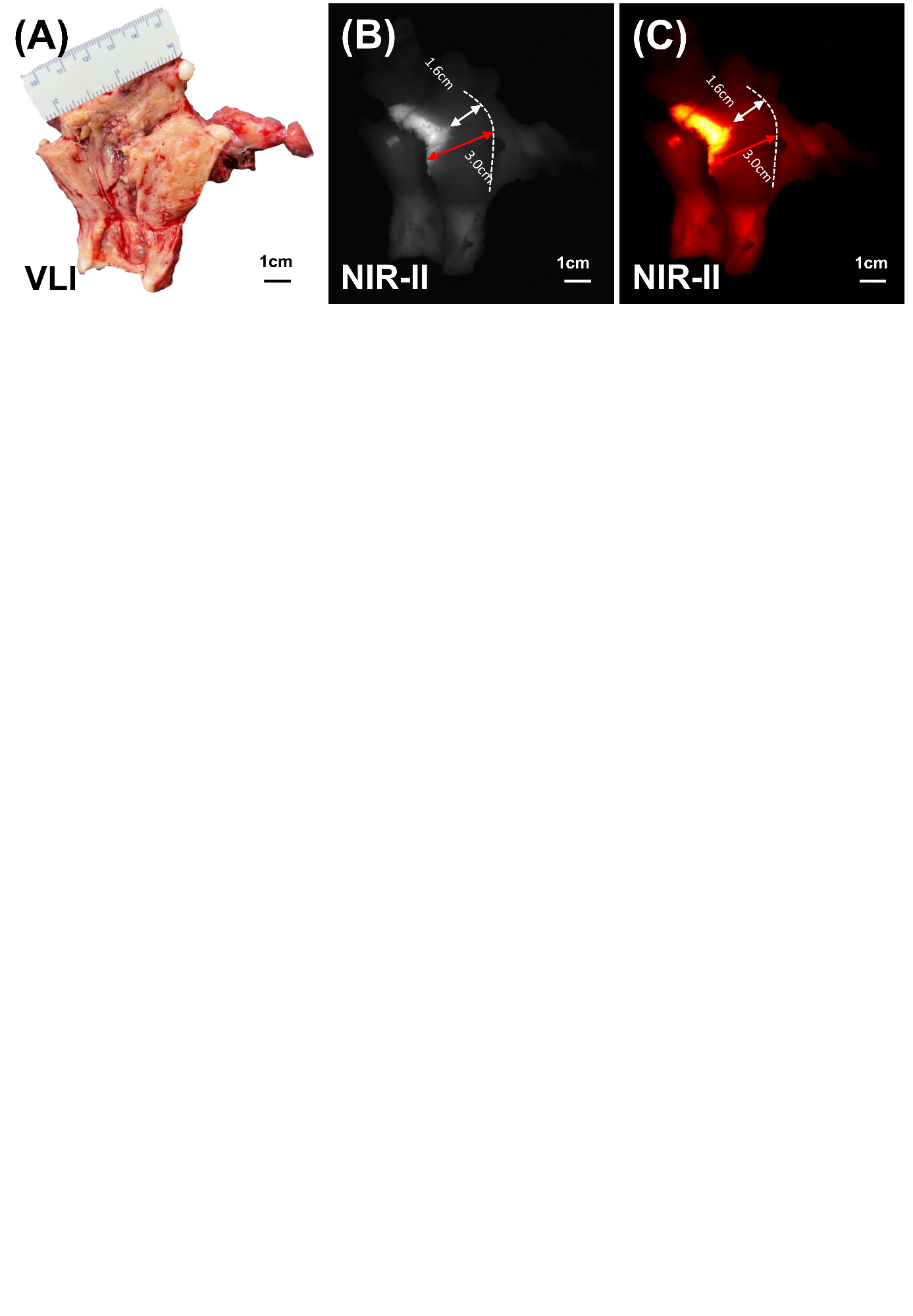


**FIGURE S3**

Assessment of MID by NIR-II fluorescence imaging. (A-C) Uninvaded and full normal myometrium thickness was measured by NIR-II fluorescence imaging.

| **Table S1 Characteristics of the study patients and lesions** | | | | | |  | |
| --- | --- | --- | --- | --- | --- | --- | --- |
| **Patient**  **(n)** | **Age**  **(years)** | **BMI** | **Pathological type** | **Histological** **grade** | **Fluorescent** | | **NIR-II**  **TBR** |
| 1 | 66 | 21.0 | endometrioid adenocarcinoma | 1 | yes | | 2.15 |
| 2 | 52 | 26.6 | endometrioid adenocarcinoma | 3 | yes | | 2.43 |
| 3 | 52 | 20.7 | endometrioid adenocarcinoma | 3 | yes | | 8.35 |
| 4 | 52 | 23.7 | endometrioid adenocarcinoma | 1 | yes | | 4.63 |
| 5 | 57 | 26.3 | endometrioid adenocarcinoma | 1 | yes | | 2.60 |
| 6 | 75 | 25.4 | endometrioid adenocarcinoma | 2 | yes | | 3.06 |
| 7 | 48 | 26.7 | endometrioid adenocarcinoma | 1 | yes | | 2.54 |
| 8 | 44 | 27.1 | endometrioid adenocarcinoma | 3 | yes | | 6.10 |
| BMI, body mass index; FIGO, International Federation of Gynecology and Obstetrics; TBR, tumor-to-background ratio. | | | | | | | |

| **Table S2 Assessment of myometrial infiltration depths** | | | | | |  |
| --- | --- | --- | --- | --- | --- | --- |
| **Patient**  **(n)** | **MRI** | **Visual observation** | **Pathology** | | **NIR-II fluorescence**  **imaging** | |
| 1 | no | ≥1/2 | ＜1/2 | | ＜1/2 | |
| 2 | ≥1/2 | ≥1/2 | ≥1/2 | | ≥1/2 | |
| 3 | no | ＜1/2 | ＜1/2 | | ＜1/2 | |
| 4 | ≥1/2 | ＜1/2 | ＜1/2 | | ＜1/2 | |
| 5 | ≥1/2 | ＜1/2 | ≥1/2 | | ≥1/2 | |
| 6 | ＜1/2 | ＜1/2 | ＜1/2 | ＜1/2 | | |
| 7 | no | ≥1/2 | ＜1/2 | ＜1/2 | | |
| 8 | ＜1/2 | ＜1/2 | ＜1/2 | ＜1/2 | | |
| MRI, magnetic resonance imaging; "no" represents no myometrial infiltration. | | | | | | |
